# Quality of Life in Alopecia Areata: A Sample of Tunisian Patients

**DOI:** 10.1155/2013/983804

**Published:** 2013-07-18

**Authors:** Jawaher Masmoudi, Rim Sellami, Uta Ouali, Leila Mnif, Ines Feki, Mariam Amouri, Hamida Turki, Abdellaziz Jaoua

**Affiliations:** ^1^Department of Psychiatry A, Hédi Chaker University Hospital, Sfax, Tunisia; ^2^Department of Dermatology, Hédi Chaker University Hospital, Sfax, Tunisia

## Abstract

*Background*. Alopecia areata (AA) has a significant impact on the quality of life and social interaction of those suffering from it. Our aim was to assess the impact of AA on the quality of life. *Methods*. Fifty patients diagnosed with AA seen in the Department of Dermatology of Hedi Chaker University Hospital, between March 2010 and July 2010, were included. Quality of life was measured by SF 36; severity of AA was measured by SALT. *Results*. Eighty percent had patchy alopecia with less than 50% involvement, 12% had patchy alopecia with 50–99% involvement, and 8% had alopecia totalis. Compared with the general population, AA patients presented a significantly altered quality of life, found in the global score and in five subscores of the SF-36: mental health, role emotional, social functioning, vitality, and general health. Gender, age, marital status, and severity of alopecia areata had a significant influence on patients' quality of life. *Conclusions*. This study indicates that patients with AA experience a poor quality of life, which impacts their overall health. We suggest screening for psychiatric distress. Studies of interventions such as counseling, psychoeducation, and psychotherapeutic interventions to reduce the impact of the disease may be warranted.

## 1. Introduction

Alopecia areata (AA) is a common disease with an incidence of 2-3% among the dermatoses and 0.1% in the population at large [1]. This disorder occurs in both sexes, at all ages [2], and is characterized by the sudden appearance of areas of hair loss on the scalp and other hair-bearing areas. Various factors, including immunologic and endocrine abnormalities [3], genetic factors [4], infections [5], and psychological/psychiatric disturbances, have been claimed to play a role in its etiopathogenesis [6]. Hence, it is suspected to be an autoimmune disease having a genetic predisposition and being influenced by environmental and ethnic factors. Epidemiological studies of AA are available from the USA, Japan, and European countries [7–9]. However, there is a paucity of data from Arab countries, with especially one published study from Kuwait done in the pediatric age group [10].

Hair loss significantly impacts an individual's self image, and studies indicate that patients with both clinically apparent and clinically imperceptible hair loss may have significantly decreased quality of life [11, 12]. Quality of life is defined as the subjective perception of the impact on the health status and on the physical, psychological, and social functioning and well-being of the patients [13]. Although AA is a medically benign condition, it can affect patients' quality of life [2]. Indeed, AA can have psychosocial complications, including depression, low self-esteem, altered self-image, and less frequent and enjoyable social engagements [14–16]. To assess the severity of AA, quality of life seems to be a more relevant criterion than clinical evaluation such as AA extension because the perception of patients may differ significantly from those of their health-care providers [13]. 

The aim of our study was to examine the impact of AA on the different dimensions of quality of life and to determine factors related to an altered quality of life. 

## 2. Methods

All new patients diagnosed with AA seen in the Department of Dermatology of Hedi Chaker University Hospital between March 2010 and July 2010 were included in the study. Informed consent was obtained from all subjects enrolled. The diagnosis of AA was made by a qualified dermatologist on a clinical basis. Sociodemographic and clinical data including age, sex, family history of AA, site of onset, and associated diseases were recorded for all patients. Included patients underwent full clinical examination to determine the number and extension of the sites affected by AA and the severity of the disease. 

The severity of hair loss was assessed by measuring the percentage of the alopecic area on the scalp. Patients with AA were evaluated using Severity of Alopecia Tool (SALT) [17]. The SALT score is computed by measuring the percentage of hair loss in each of 4 areas of the scalp (40% vertex, 18% right profile, 18% left profile, 24% posterior) and adding the total to achieve a composite score. 

Patients were divided into four groups according to disease severity: S1-S2: hair loss below 50%; S3-S4: hair loss of 50–99%; S5: total scalp hair loss.

Following examination, a health survey in its short form (SF36) was administered to all included patients. The SF36 health survey is a widely used generic, 36-item, self-reported health status questionnaire assessing eight domains of health status (1) physical functioning; (2) role physical; (3) bodily pain; (4) mental health; (5) role emotional; (6) social functioning; (7) vitality; (8) general health. A score from 0 to 100 is calculated for each subscale, with higher scores indicating better quality of life. It has been translated into Arabic and validated but not yet published [18]. Physical functioning domain addresses physical activities associated with daily life, such as bathing, walking, or carrying groceries. Role-physical domain assesses how physical health affects work. Bodily pain domain measures pain severity and how it interferes with daily activities. Mental health domain assesses the subject's mood, specifically focusing on feelings of sadness and anxiety. Role emotional domain addresses how the subject's emotional state has influenced work and other daily activities. Social functioning domain measures how much emotional or physical problems have interfered with usual social activities. Vitality domain assesses how energetic or tired the subject feels. Finally, general health domain describes how the patient perceives health status. The findings of the SF-36 were compared with those of a control group consisting of healthy volunteers, with no known chronic skin disease, of similar age, sex, and level of education. The group of patients and 50 controls did not show significant differences concerning age (*P* = 1.000), educational level (*P* = 0.802), and gender (*P* = 0.987). The control group had not any organic or psychiatric disease.

The mean total score of each domain of SF36 in patients with AA was compared with the mean score of the control group by *t*-test. The *t*-test comparisons for each subscale were evaluated with statistical significance designated as *P* less than 0.05. We also examined the relationship between different domains of quality of life and several independent variables, including marital status, gender, illness severity, and age. Associations with quality of life were tested using *t* tests and one-way ANOVAs with post hoc LSD multiple comparison tests and correlations (Spearman and Pearson). The use of parametric versus nonparametric tests was dependent on the scale of measurement and distribution of the results.

## 3. Results

Fifty patients were included in the study. Their mean age was 32.92 years (SD = 11.81), having a minimum of 18 years and a maximum of 60 years. There were 48% males and 52% females with a male to female ratio of 0.92. As to the level of education, 18% had elementary school education, 40% had secondary school education, and 42% had higher education level.

Fifty-two percent of patients were single, 46% were married, and 2% of them were divorced. As to occupation, most worked (52%) some were retired (2%), and whereas the others were studying (24%) or unemployed (22%). 

At the time of first presentation, 80% had patchy alopecia with less than 50% involvement (S1-S2), 12% had patchy alopecia with 50–99% involvement (S3-S4), and 8% had alopecia totalis. 

Patient's quality of life, demonstrated by SF-36 scores, ranged from 38.54 to 92.7 with a mean 68.95 (±13.10). Compared with the general population (Table 1), AA patients presented a significantly altered quality of life, found in the global score and in five subscores of the SF-36: mental health (D4), role emotional (D5), social functioning (D6), vitality (D7), and general health (D8) (Table 1).

Females scored lower on the SF 36 than males: this gender difference was significant for the global score (*P* = 0.007) and three subscores: physical functioning (*P* = 0.028), general health (*P* = 0.012), and role emotional (*P* = 0.018). 

The correlation of Pearson indicated a significantly altered mental health domain in younger patients (*r* = 0.336, *P* = 0.017). 

Furthermore, we observed a significant difference in the scores of the mental health subscale (D4) between married and unmarried patients (*P* = 0.050): unmarried patients scored lower than married patients. 

In our study, we found a relationship between poorer quality of life and severity of AA. This relationship was significant in the SF-36 subscale of mental health and social functioning: patients with 51% to 75% of hair loss (S3) showed significantly lower scores on the social functioning subscale compared to those with hair loss less than 25% (S1) (Table 2). Patients with 100% extent of hair loss (S5) showed significantly lower scores on the mental health subscale compared to those with 51% to 75% of hair loss (S3). Severity of AA was related to lower scores in the mental health domain (*r* = −0.379; *P* = 0.007) as well as lower scores in the social functioning domain (*r* = −0.365; *P* = 0.009). 

## 4. Discussion 

In our study, we observed a slight female preponderance for AA. (M/F: 0.92). This is in agreement with other studies [19–21]. However, the results of the literature are disparate. Thus, for some authors, AA affects men and women with the same proportions [2, 22, 23]. 

We have found that included patients had a lower quality of life compared to the control group. Dimensions of the SF36 referring to psychological and social components were particularly affected. Indeed, several studies underline that the experience of AA is psychologically damaging, causes intense emotional suffering, and leads to personal, social, and work related problems [24]. Our results are in line with authors' findings that report quality of life to be seriously impaired, mainly, by altering self-perception and self-esteem [25, 26]. 

This may be because of the special importance of hair in appearance [27]. Hair is a distinctive and valued facial characteristic, and the way it is styled helps to define the individual's self-concept and identity. Through hair loss, the ability to manipulate and improve appearance may become uncertain and out of one's control [28, 29]. In fact, AA might make the individual focus on the bold patches, which leads to even greater distress.

On the other hand, the alteration of psychological and relational dimensions of patients' quality of life could be dependent on the regular recurrence and unpredictable severity of AA, leaving a feeling of incomprehension and vulnerability [30]. Moreover, the unpredictable hair loss and the fact that there is currently no effective remedy could cause particular anxiety related to uncertainty about the patient's future appearance [31]. 

The disease not only has a severe psychological impact but also causes a marked disturbance in the social life of the patients forcing them to avoid social meetings, change their hair style, and alter the type of clothing. In Tunisia, where Islamic customs prevail, women try to cover their hair. This observation was found in Kuwaiti and Egyptian studies [32]. 

Our results showed that women have a more significantly altered quality of life than men. The dimensions of general health and role emotional were particularly affected. In consistency with our findings, several other studies have reported a particularly impaired quality of life for women [11, 33]. Femininity, sexuality, and personality are symbolically linked to a woman's hair, more so than for a man [34]. Indeed, a women's self-esteem is often more dependent on physical appearance compared to men [29], and hair strongly influences whether or not an individual is seen as physically attractive [35]. In line with social stereotypes, men were more likely to express concern over work interference, whereas women were more concerned with the social impact of such a visible condition. There is an important link between hair and identity, especially for women, even in Arab Muslim culture. Hair loss may be seen in terms of abnormality and as a failure to conform to the norms of physical appearance in society, which has the potential to set people apart in their own estimation and in the estimation of others. So, AA in women significantly affects self-image, and psychosocial factors often negatively impact patients' quality of life [33, 36–38]. 

In our study, we found that a patient's age was correlated to the SF 36 dimension of mental health: the younger the patient is, the more his mental health is negatively affected by AA. Furthermore, our study showed that unmarried patients had significantly altered scores in the dimension of mental health compared to married patients. Our results are in accordance with two recent studies [32, 38] which found that the impact of AA on the quality of life was more pronounced within younger age groups. Indeed, hair loss would entail more concern among those most vulnerable such as younger patients. These results denote that unestablished social life in younger and unmarried patients makes them worry about their future resulting in more psychological distress.

**Figure 1:**
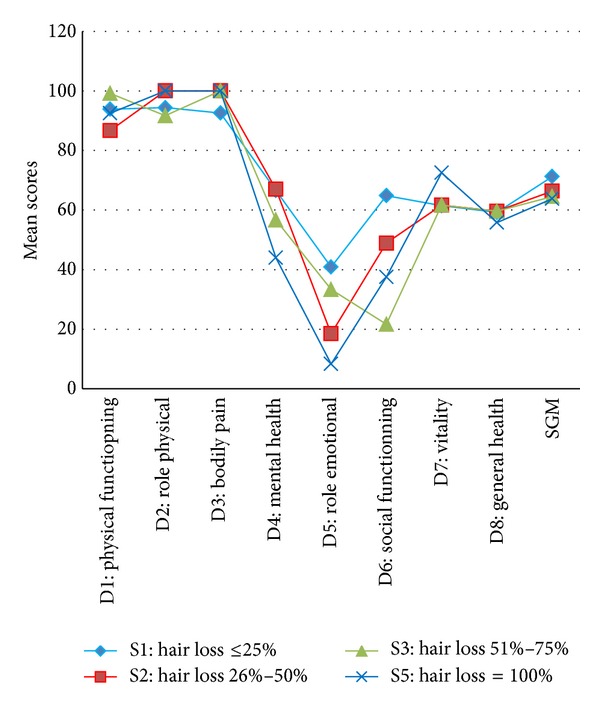
Mean scores SF-36 depending on the severity (SALT).

In our study, we found a relationship between the severity of AA and poorer quality of life (Figure 1). 

The domains referring to psychological and social components were particularly affected in patients with severe forms of AA. Indeed, given the psychological importance of hair, patients with severe forms of AA might find it more difficult to cope with their hair loss. According to Tan et al. [19], patients who presented limited AA appeared to be less affected than those who had an extensive AA. This shows that if AA is more severe, its impact on quality of life is more important. However, the relationship between disease severity and impaired quality of life has not been confirmed by all studies [39]. A study by Krueger et al. [40] revealed that AA areas in particularly visible locations had a more severe impact on quality of life than an area of the same size in a less visible location. Interestingly, a study by Reid et al. [41] showed that patients' quality of life was strongly correlated with the patient's perception of hair loss rather than with the clinical severity of hair loss. Generally, patients tend to perceive their hair loss as worse than clinical assessment, but patients and physicians seem to have synchronous scales of severity. 

This lack of parallelism between clinical severity, patients' perception of hair loss, and psychological impact might be partly responsible for the frequent underestimation of psychological distress [42]. 

There are some limitations to our study that should be taken into consideration. First, the sample size was relatively small. Furthermore, it could have been interesting to assess the quality of life with a scale specially designed for dermatologic patients. However, to our knowledge, there is no such instrument translated in Arabic language so far. 

## 5. Conclusion

This study indicates that patients with AA experience a poor quality of life, which profoundly impacts their overall health and sense of well-being. Also, when examining a patient who is at high risk for poor quality of life, it would be reasonable to screen for psychiatric distress. Those with mild impairments should be encouraged to discuss their concerns at clinic visits or in a support-group setting. Moreover, the efficacy of any treatment must also consider the improvement in quality of life and not just see the percentage of hair regrowth. Studies of interventions such as counseling, psychoeducation, and psychotherapeutic interventions to reduce the impact of the disease may be warranted.

## Figures and Tables

**Table 1:** Quality of life comparisons between AA patients and controls.

Dimensions of SF-36	Patients M ± ET	Controls M ± ET	*P *
D1: physical functioning	93.10 ± 12.45	88.30 ± 17.57	0.118
D2: role physical	95.50 ± 10.93	90.20 ± 16.31	0.059
D3: bodily pain	95.40 ± 12.48	89.80 ± 16.22	0.056
D4: mental health	63.64 ± 16.61	77.14 ± 11.03	<0.001
D5: role emotional	33.33 ± 36.26	83.06 ± 24.40	<0.001
D6: social functioning	54.60 ± 33.75	82.20 ± 18.56	<0.001
D7: vitality	62.40 ± 21.19	77.00 ± 14.70	<0.001
D8: general health	58.17 ± 17.06	71.62 ± 13.71	<0.001
Mean score	68.95 ± 13.10	80.52 ± 9.14	<0.001

**Table 2:** Correlation between quality of life and severity.

Dimensions of SF-36	S1 M ± ET	S2 M ± ET	S3 M ± ET	S5 M ± ET	ANOVA	
					*F *	*P *
D1: physical functioning	93.87 ± 10.3	86.66 ± 21.06	99.16 ± 2.04	92.5 ± 8.66	1.347	0.271
D2: role physical	94.35 ± 10.62	100	91.66 ± 20.41	100	1.099	0.359
D3: bodily pain	92.58 ± 15.26	100	100	100	1.422	0.249
D4: mental health	66.54 ± 13.93	67 ± 15.65	56.66 ± 20.61	44 ± 21.66	2.978	0.041
D5: role emotional	40.85 ± 38.2	18.51 ± 33.79	33.33 ± 29.81	8.33 ± 16.66	1.641	0.193
D6: social functioning	64.83 ± 29.19	48.88 ± 30.18	21.66 ± 32.5	37.5 ± 43.49	3.856	0.015
D7: vitality	61.45 ± 19.67	59.62 ± 12.07	61.66 ± 32.5	72.5 ± 5	0.316	0.814
D8: general health	59.21 ± 16.65	66.37 ± 13.33	52.2 ± 26.35	55.83 ± 17.92	0.315	0.815
Mean score	71.22 ± 13.33	80.52 ± 9.14	64.54 ± 14.45	63.82 ± 12.09	0.850	0.474
